# Hydrophobicity-Driven Enhancement of Hydrogen Bonding in Ionic Liquid Hybrid Solvents

**DOI:** 10.1021/acs.jpcb.5c01687

**Published:** 2025-07-04

**Authors:** Samuel Abidemi Oluwole, Welday Desta Weldu, Christian Agatemor

**Affiliations:** † Department of Chemistry, 5452 University of Miami, Coral Gables, Florida 33146, United States; ‡ Department of Chemistry, 4517 Bucknell University, Lewisburg, Pennsylvania 17837, United States

## Abstract

Intermolecular interactions play a fundamental role in the properties of materials, including solvents. Yet, the complex interplay between these interactions, specifically hydrophobic interactions and hydrogen bonding (H-bonding), in liquids, such as the emerging class of ionic liquid hybrid solvents (ILHSs), where these interactions coexist, remains to be fully grasped. Understanding these interactions is crucial for designing new ILHSs and optimizing their performance. Here, we used orthogonal techniques to elucidate the interplay between hydrophobicity and H-bonding in ILHSs composed of ionic components, choline and geranate, and a terpenoid molecular solvent. Our findings reveal a counterintuitive strengthening of H-bond networks with increasing molecular hydrophobicity, uncovered through integrated spectroscopic, calorimetric, and DFT analyses. This insight provides a new framework for understanding how molecular-scale hydrophobicity modulates mesoscopic organization in complex fluids and structured solvents, with implications for enhanced nanoscale organization, biologic encapsulation, and the rational design of ILHSs for applications such as catalysis and drug delivery.

## Graphical abstract

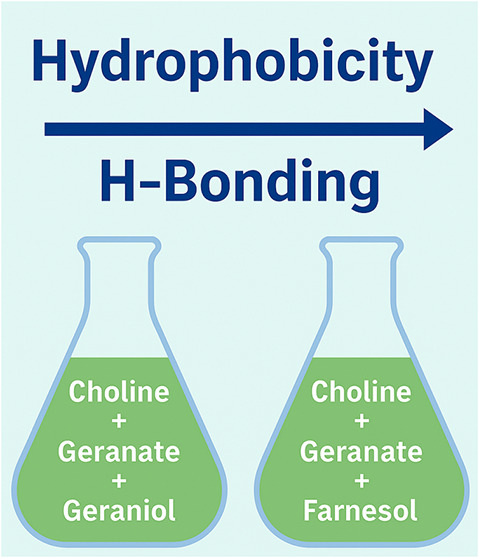

## 1 Introduction

Ionic liquids (ILs) are a promising class of solvents considered tunable and sustainable alternatives to conventional laboratory solvents.^1–7^ Despite their promise, several issues, such as high viscosity and hygroscopicity, limit their performance in some applications. Recently, researchers have circumvented these limitations by designing ionic liquid hybrid solvents (ILHSs) derived by combining ILs with molecular solvents.^8–27^ For instance, combining 1-butyl-3-methylimidazolium tetrafluoroborate, an archetypal IL, with molecular solvents, such as methanol or *N*-methyl-2-pyrrolidone, yields ILHSs with reduced viscosity.^15^ Similarly, combining an IL composed of a 1:1 molar ratio of choline and geranate with geranic acid resulted in CAGE, an ILHS with reduced viscosity and enhanced lipophilicity, enabling applications in drug delivery.^8–14,28–30^ Indeed, ILHSs are emerging as a versatile class of solvents with properties capable of advancing diverse fields, ranging from biomedicine to chemical synthesis.

A critical factor influencing the ILHS functionality is the intricate balance between the different intermolecular interactions within the solvent. Typically, such balance dictates all solvents’ properties, including viscosity, miscibility, and reactivity.^31–33^ For example, the unique properties of ILs originate from an interplay between the Coulombic interaction and other interactions such as hydrogen bonding (H-bonding), van der Waals, π–π interactions, and dipole–dipole interactions.^34–45^ In prior works, we and others postulated that Coulombic interactions and H-bonding are pivotal to the overall behavior of CAGE, an ILHS mentioned above.^11,46^ Nevertheless, critical gaps in knowledge remain regarding the interplay between these interactions in ILHSs and solvents where multiple interactions coexist. Specifically, a fundamental understanding of the interplay between hydrophobic interactions and H-bonding within ILHSs remains unclear. Bridging this gap will pave the foundation for the rational design of the next generation of ILHSs and for optimizing their performance during diverse applications. This work aims to elucidate the interplay between hydrophobicity and hydrogen bonding in ILHSs. Toward this, we designed CAGE-type ILHSs composed of choline, geranate, and a terpenoid molecular solvent, specifically geraniol or farnesol (Figure 1a,b), as a model due to their ability to engage in H-bonding and hydrophobic interactions. The rationale for selecting these CAGE-type ILHSs is that their structural feature allows coexistent hydrophobic and H-bonding interactions. Specifically, they feature carboxylate, carboxyl, and hydroxyl groups that enable strong H-bonding formation and a hydrocarbon alkenyl tail that contributes to hydrophobic interactions. We used orthogonal techniques to probe the interplay between hydrophobicity and H-bonding and explored the ILHSs’ ability to stabilize ribonucleic acid (RNA).

**Figure 1:**
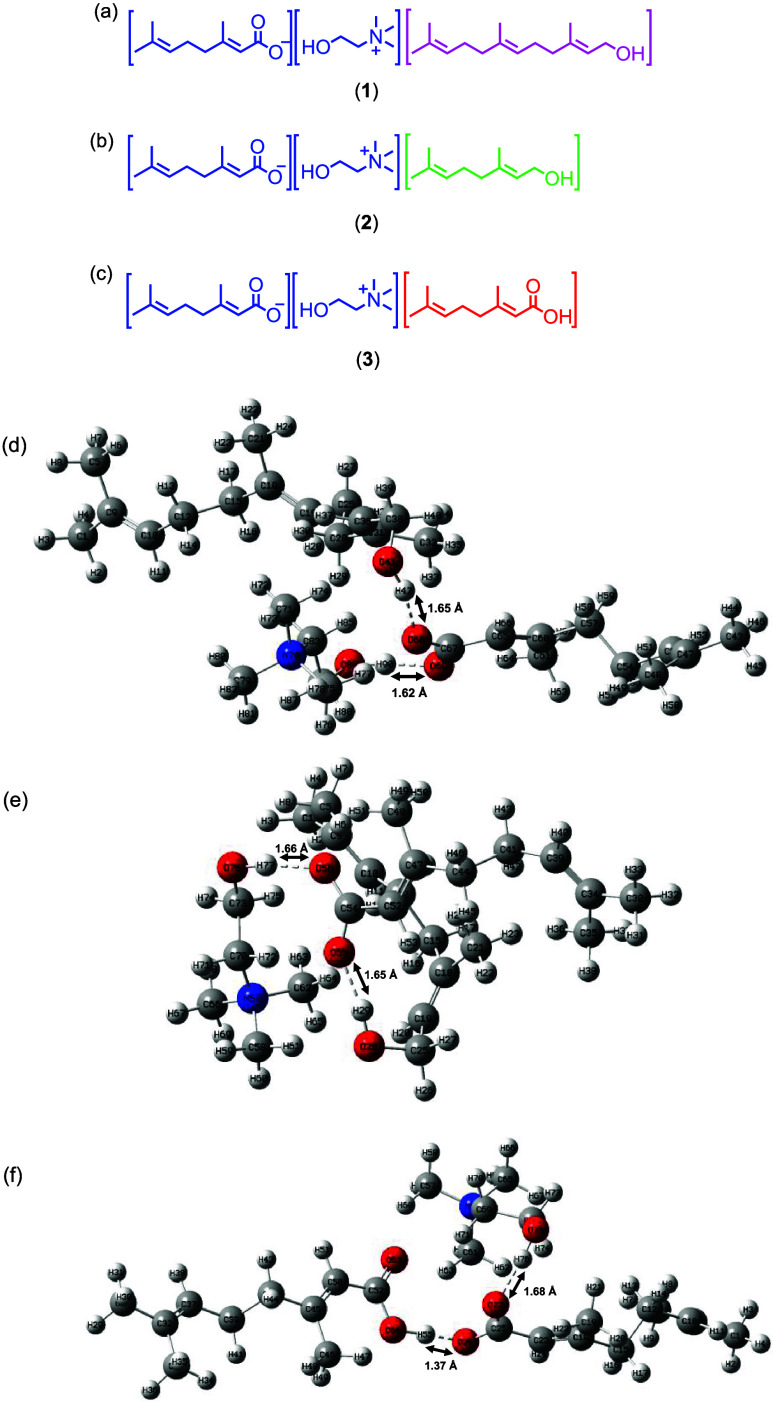
(a–c) Structural representation of the ILHSs. An ILHS is composed of an ionic liquid (blue) derived from cholinium cation and geranate anion and a molecular solvent—farnesol (purple (**1**)), geraniol (green (**2**)), or geranic acid (red (**3**)). (d–f) DFT-calculated H-bond lengths based on the B3LYP function and 6-311G basis set optimized structures of **1**–**3**. (d) Optimized structure of **1**; (e) optimized structure of **2**; (f) optimized structure of **3**. Red is oxygen atoms, blue is nitrogen atoms, gray is hydrogen atoms, and black is carbon atoms.

## 2 Experimental Methods

### 2.1 Materials

Deuterated dimethyl sulfoxide (DMSO), choline bicarbonate, geraniol, farnesol, and geranic acids were purchased from Sigma-Aldrich, and mCherry fluorescent protein was purchased from antibodies-online. The mCherry RNA was obtained from APExBIO Technology LLC. All of the materials were used directly except geranic acid, which was recrystallized from acetone three times based on a previously described protocol.^8^

### 2.2 Ionic Liquid-Based Hybrid Solvent (ILHS) Synthesis

The three ILHSs (**1**–**3**) were synthesized using a previously reported protocol.^8^ For example, **1** was synthesized by reacting one mol of choline bicarbonate, one mol of geranic acid, and one mol of farnesol. The reaction mixture was stirred for 3 h, during which time CO_2_ effervescence was completed. Water was removed using a rotary evaporator for 20 min at 60 °C and 72 mbar. The resulting solutions were placed in the vacuum oven for 48 h at 60 °C to remove the residual water. The ILHSs were characterized using ^1^H, ^13^C, and HSQC NMR spectroscopy (Figures S1–S9).

### 2.3 Instrumentation and Sample Characterization

For NMR spectroscopic characterization (Figures S1–S9), the ILHS was placed in the outer tube of a Wilmad coaxial insert with DMSO-*d*_6_ placed in the inner tube. The 1D and HSQC NMR experiments were conducted by using a Bruker Avance 800 MHz spectrometer. For variable-temperature ^13^C NMR, the temperature was set at 25 or 0 °C, and the experiment was run on a Bruker Avance 500 MHz spectrometer. The infrared (IR) spectroscopy experiment was performed using a PerkinElmer FT-IR spectrometer Frontier instrument. The samples were placed on the instrument sample holder, and the readings were taken accordingly. Thermogravimetric analysis (TGA) was conducted using a Netzsch TG 209 F3 Tarsus with a sample (50 mg) placed in an Al_2_O_3_ crucible and heated from 25 to 700 °C at a heating rate of 10 °C/min under nitrogen. Differential scanning calorimetry (DSC) was conducted using Discovery DSC 2500 (TA Instruments) with the sample placed in a Tzero aluminum hermetic pan under nitrogen (50 mL/min) using a heat–cool–heat cycle at a temperature ramp rate of 5 °C/min. Dynamic light scattering (DLS) analysis was performed using a Malvern Zetasizer ultra, optimized for each sample condition, including accumulations, before collecting the data. A 50 mM micellar solution of ILHS was prepared in water for the DLS experiment. To encapsulate mCherry in the ILHS micelles, the micellar solution was spiked with the mCherry protein to a concentration of 5 μg/mL (174 nM). Small-angle X-ray scattering (SAXS) experiments were performed by using the SAXSlab Ganesha instrument. A Xenocs GeniX3D microfocus source was used with a Cu target to generate a monochromatic beam with a 0.154 nm wavelength. The instrument was calibrated using the National Institute of Standards and Technology (NIST) reference material 640d silicon powder with the peak position at 2θ = 28.44° where 2θ is the total scattering angle. A Pilatus 300 K detector (Dectris) was used to collect 2D scattering patterns with pixel dimensions of 172 × 172 μm^2^. The SAXS data was acquired with an X-ray flux of ∼16.1 million photons per second incident upon the sample and with a sample-to-detector distance of 440 mm. The 2D images were azimuthally integrated to determine the scattering vector intensity. The peak positions were fitted by using custom Matlab software. For the RNA stabilization experiment, a cocktail containing mCherry RNA (5 μg/μL) in 50 mM ILHS was incubated at room temperature, while controls, having RNA (5 μg/μL) in water, were incubated at room temperature, 4 °C, or −80 °C. The RNA stability was monitored for 7 days using agarose gel electrophoresis on E-Gel Power Snap Plus Electrophoresis Systems (Invitrogen). Aliquots from the cocktail were diluted 50-fold in water before running the electrophoresis based on the instrument manufacturer’s instructions, and the gel was imaged using the iBright 1500 imaging system model FL1500 (ThermoFisher Scientific).

### 2.4 Computational Methodology

All density functional theory (DFT) calculations were performed using Gaussian 16 on the University of Miami Pegasus supercomputing cluster. The B3LYP exchange–correlation function was employed with the 6-311G basis set, which provides a suitable balance between computational cost and accuracy for small to medium-sized molecular systems. Initial cluster geometries were constructed by using GaussView (version 6.1.1). Each cluster contained one choline (HBA and HBD), one geranate (HBD), and one molecular solvent (farnesol, geraniol, or geranic acid) (HBA and HBD), corresponding to ILHS systems **1**–**3** investigated in this study. The HBA and HBD components were arranged to favor strong H-bonding using idealized D–H···A distances of ∼2.5 Å and bond angles near 180°. The molecular solvent tail was the only structural variable among the three systems. For each ILHS, multiple cluster configurations were constructed by varying the orientation and placement of the molecular solvent (HBA and HBD) and choline (HBA and HBD) relative to geranate (HBD). These configurations were independently optimized using full unconstrained geometry optimizations at the B3LYP/6-311G level. All atoms were allowed to relax, and vibrational frequency analysis confirmed the absence of imaginary frequencies, implying that the resulting structures corresponded to the true local minima. Among the optimized clusters for each ILHS, only the lowest-energy structure was selected for further analysis. Although these optimized clusters represent only local minima and do not model the full complexity of the bulk ILHS liquid phase, they enable consistent comparisons of H-bond distances across **1**–**3**. Notably, although the hydrophobic tails do not interact in these minimal clusters, their steric and electronic properties should influence the spatial distribution and electron density near the H-bonding domains, affecting the strength of the H-bond.

### 2.5 NMR Spectroscopic Data of **1**

^1^H NMR (800 MHz, CDCl_3_): δ (ppm) 5.58 (CH), 5.35 (CH), 5.07–5.03 (CH, CH, CH), 4.06 (CH_2_), 3.95 (CH_2_), 3.50 (CH_2_), 3.18 (C­(CH_3_)_3_), 2.06–1.93 (CH_2_, CH_2_, CH_2_, CH_2_, CH_2_, CH_2_, CH_3_), 1.69–1.54 (CH_3_, CH_3_, CH_3_, CH_3_, CH_3_) ppm. ^13^C NMR (800 MHz, CDCl_3_): δ 174.42 (C=O), 147.26 (C), 139.08 (C),135.37 (C), 131.70 (C), 124.83–123.74 (C, CH, CH, CH, CH, CH), 68.04 (CH_2_), 59.09 (CH_2_), 55.92 (CH_2_), 54.25 (3x CH_3_), 40.47 (CH_2_), 39.98 (CH_2_), 39.79 (CH_2_), 39.70 (CH_2_), 32.06 (CH_2_), 26.82–26.33 (CH_2_), 25.78 (CH_3_), 23.47 (CH_3_), 18.05 (CH_3_), 17.77 (CH_3_), 16.35 (CH_3_), 16.08 (CH_3_) ppm.

### 2.6 NMR Spectroscopic Data of **2**

^1^H NMR (800 MHz, CDCl_3_): δ 5.56 (CH), 5.31 (CH), 5.03–5.01 (CH, CH), 4.05 (CH_2_), 3.93 (CH_2_), 3.48 (CH_2_), 3.17 (C­(CH_3_)_3_), 2.04–1.92 (CH_2_, CH_2_, CH_2_, CH_2_, CH_3_), 1.62–1.52 (CH_3_, CH_3_, CH_3_, CH_3_, CH_3_) ppm. ^13^C NMR (800 MHz, CDCl_3_): δ 174.28 (C=O), 147.01 (C), 138.54 (C), 131.56 (C), 124.01–123 (CH), 67.90 (CH_2_), 58.79 (CH_2_), 55.76 (CH_2_), 54.12 (3x CH_3_), 40.53 (CH_2_), 39.57 (CH_2_), 26.52 (CH_2_), 26.43 (CH_2_), 25.63 (CH_3_), 17.91 (CH_3_), 17.64 (CH_3_), 17.63 (CH_3_) ppm.

### 2.7 NMR Spectroscopic Data of **3**

^1^H NMR (800 MHz, CDCl_3_): δ (ppm) 5.58 (CH), 5.01 (CH), 3.93 (CH_2_), 3.48 (CH_2_), 3.17 (C­(CH_3_)_3_), 2.02–1.95 (CH_2_, CH_2_, CH_3_), 1.60–1.52 (CH_3_, CH_3_) ppm. ^13^C NMR (800 MHz, CDCl_3_): δ 173.45 (C=O), 149.76 (C), 131.81 (C), 123.83 (CH), 122.40 (CH), 68.04 (CH_2_), 55.93 (CH_2_), 54.26 (3x CH_3_), 40.53 (CH_2_), 26.95 (CH_2_), 25.73 (CH_3_), 18.21 (CH_3_), 17.74 (CH_3_) ppm.

## 3 Results and Discussion

Hydrophobicity influences the degree and dynamics of H-bonding in solvents.^47–52^ In traditional solvents, for instance, hydrophobic interactions weaken or strengthen H-bonding networks depending on molecular context.^53,54^ In ILHSs, depending on the components, hydrophobic interactions can coexist with other interactions, such as Coulombic interactions and H-bonding, creating a scenario for a convoluted interplay between these interactions. Currently, the dynamics of this interplay remains to be fully understood, prompting the present study. To elucidate the dynamics, we designed a novel ILHS (**1**) by combining farnesol, a molecular solvent composed of hydrophobic 15-carbon terpene alcohol, with an IL consisting of a 1:1 mixture of choline and geranate ions (Figure 1a). The expectation is that farnesol will enhance the hydrophobicity of the resulting ILHS. This design principle builds on previous reports where the hydrophobicity of deep eutectic solvents, neoteric solvents akin to ILs, originates from hydrophobic components.^55–59^ We synthesized **2** (Figure 1b) based on the same principle by replacing farnesol with geraniol, a hydrophobic 10-carbon terpene alcohol. These ILHSs have the same H-bond donors (HBDs) and acceptors (HBAs), but **1** will be more hydrophobic than **2** due to farnesol’s superior hydrophobicity/lipophilicity (log *K*_ow_: 5.77) over geraniol (log *K*_ow_: 3.56).^60^ Therefore, investigating **1** versus **2** will unravel how increasing hydrophobicity impacts H-bonding in ILHSs. To decouple the effects of H-bonding from alkenyl chain length (molecular size), we employed the previously reported CAGE, **3**,^8–14^ which incorporates geranic acid—a hydrophobic 10-carbon carboxylic acid structurally analogous to geraniol in **2** in chain length but distinct in H-bonding capacity (Figure 1c)—as a control. While compounds **2** and **3** possess comparable alkenyl chain lengths, their ability to form H-bonds differs. This molecular design should isolate the role of H-bonding without confounding differences in chain length or overall hydrophobicity.

Building on the known correlation between bond length and strength, we use Gaussian 16-based density functional theory (DFT) calculations^61^ to compute the H-bond lengths between the carboxylate oxygens (HBAs) in geranate and the hydroxyl hydrogens (HBDs) in choline, geraniol, and farnesol or the carboxyl hydrogen (HBD) in geranic acid (Figure 1). These experiments used GaussView to construct multiple clusters of **1**, **2**, or **3** with the components positioned in a geometry where the donor (D–H) and acceptor (A) are aligned in space (D–H···A distance = ∼2.5 Å and D–H···A angle = ∼180°) to allow for H-bonding.^62–66^ The DFT analyses were based on the B3LYP/6–311G basis set optimized (lowest energy) structures of **1**–**3** and revealed a striking H-bond length pattern. The H-bonding between hydroxyl hydrogens of farnesol in **1** or geraniol in **2** and carboxyl oxygen in geranate exhibited the same H-bond lengths (1.6518 Å) (Figure 1d,e). Although farnesol is more hydrophobic than geraniol, the identical H-bond lengths indicate that the hydrophobicity of the molecular solvents does not strongly influence its H-bonding interaction with geranate, an ionic component of the ILHS. Nevertheless, increased hydrophobicity of the molecular solvent enhances the H-bonding interaction between the choline cation and geranate anion. Indeed, counterintuitively, the H-bond lengths between the hydroxyl hydrogen of choline and the carboxyl oxygen of geranate in **1** (1.6198 Å) were shorter than in **2** (1.6624 Å), despite the increased hydrophobicity introduced by farnesol (Figure 1d,e). Farnesol, being more hydrophobic than geraniol, may better structure the local organization of the ILHS, bringing the polar, ionic moieties, choline, and carboxylate headgroup of geranate, in proximity, resulting in a shorter H-bond length. Also, increasing hydrophobicity can force polar H-bonding groups into fewer orientations (lower entropy), strengthening the H-bonding networks. Solvents like ILs and ILHSs exhibit microheterogeneity with phase-separated polar and nonpolar domains.^9,67–70^ Therefore, we hypothesize that increasing the alkenyl chain length brings the polar group together or confines them into a few orientations within the polar domains to strengthen H-bonding.

Noteworthily, increasing the H-bonding ability of the molecular solvent by replacing geraniol in **2** with geranic acid in **3** enhances its H-bonding interaction with the geranate but attenuates the interaction between the choline and geranate. Evidently, in **3**, the H-bond length between the geranic acid carboxyl hydrogen and a geranate oxygen was the shortest (1.3660 Å), while that between the choline hydroxyl hydrogen and the other geranate oxygen was the longest (1.6800 Å) in the series (Figure 1f). This trend is expected since the carboxyl group in geranic acid (**3**) is more capable of forming a H-bond than the hydroxyl group in farnesol (**1**) and geraniol (**2**). On the other hand, the extended nature of the H-bond length between choline and geranate in **3** was intriguing. Still, it could be attributed to two factors: (1) steric hindrance imposed by the carboxyl group, which could disrupt the proximity between choline and carboxylate headgroup of geranate, increasing the H-bonding length and/or (2) resonance effect in the carboxylate headgroup of geranate, which can redistribute electron density after a strong H-bonding with geranic acid, weakening H-bonding with choline. Overall, the DFT computation suggests that the hydrophobicity and H-bonding ability of the molecular solvents can influence the H-bonding interaction between H-bonding moieties of the ILHS. Also, it is important to note that these optimized structures are local minima rather than global or bulk-phase configurations. Still, they enable consistent comparisons of H-bonding across the systems, **1**–**3**. Although the hydrophobic tails do not directly interact in these minimal clusters (Figure 1d–f), it is rational to assume that they still shape the spatial and electronic environment of the H-bonding functional groups, subtly tuning the H-bond length.

DFT-calculated H-bond lengths can provide helpful insights into H-bond strength, but these insights are incomplete. Therefore, to corroborate the DFT calculations, we used orthogonal techniques—attenuated total reflectance infrared (ATR-IR) spectroscopy, differential scanning calorimetry (DSC), thermogravimetry analysis (TGA), small-angle X-ray scattering (SAXS), and variable-temperature ^13^C nuclear magnetic resonance spectroscopy (VT ^13^C NMR) to probe the interplay between hydrophobicity and H-bonding in the ILHSs. ATR-IR spectroscopy provides information about H-bonding through changes in vibrational band wavenumbers, intensity, and shape. Usually, H-bonding shifts the band to lower wavenumbers, broadens the band, or increases the band intensity of H-bonding groups such as hydroxyl (OH) and carbonyl (CO).^71–75^ ATR-IR spectroscopic analyses of the ILHSs corroborated the DFT calculations, indicating that hydrophobicity strengthened H-bonding. Specifically, the OH stretching band maximum intensity blue-shifted from 3235 to 3243 cm^–1^, and the band intensity decreased upon replacing the more hydrophobic farnesol in **1** with less hydrophobic geraniol to form **2** (Figure 2a). Expectedly, the ATR-IR analyses also indicate that the wavenumber of the OH stretching band maximum intensity red-shifted from 3243 to 3220 cm^–1^ with a concomitant increase in band intensity upon replacing geraniol in **2** with geranic acid to form **3**, suggesting strengthened H-bonding (Figure 2a). Notably, although DFT predicts stronger H-bonding between geranic acid and geranate and weaker interactions between choline and geranate in **3**, this site-specific trend is not clearly resolved in the ATR-IR spectrum. This is not a contradiction between the techniques but rather a reflection of their fundamental differences. DFT provides detailed, localized insights into individual H-bonding interactions within isolated molecular clusters, whereas ATR-IR captures ensemble-averaged vibrational signals from all OH groups in the bulk liquid. In **3**, the OH stretching bands of choline and geranic acid overlap significantly, making it difficult to deconvolute the contributions of each to the overall H-bonding pattern. Thus, what appears as a discrepancy is in fact a difference in technique resolution and perspective. Together, DFT and ATR-IR offer complementary information, with DFT helping to interpret subtle molecular-level interactions that are otherwise masked in bulk-phase measurements.

**Figure 2:**
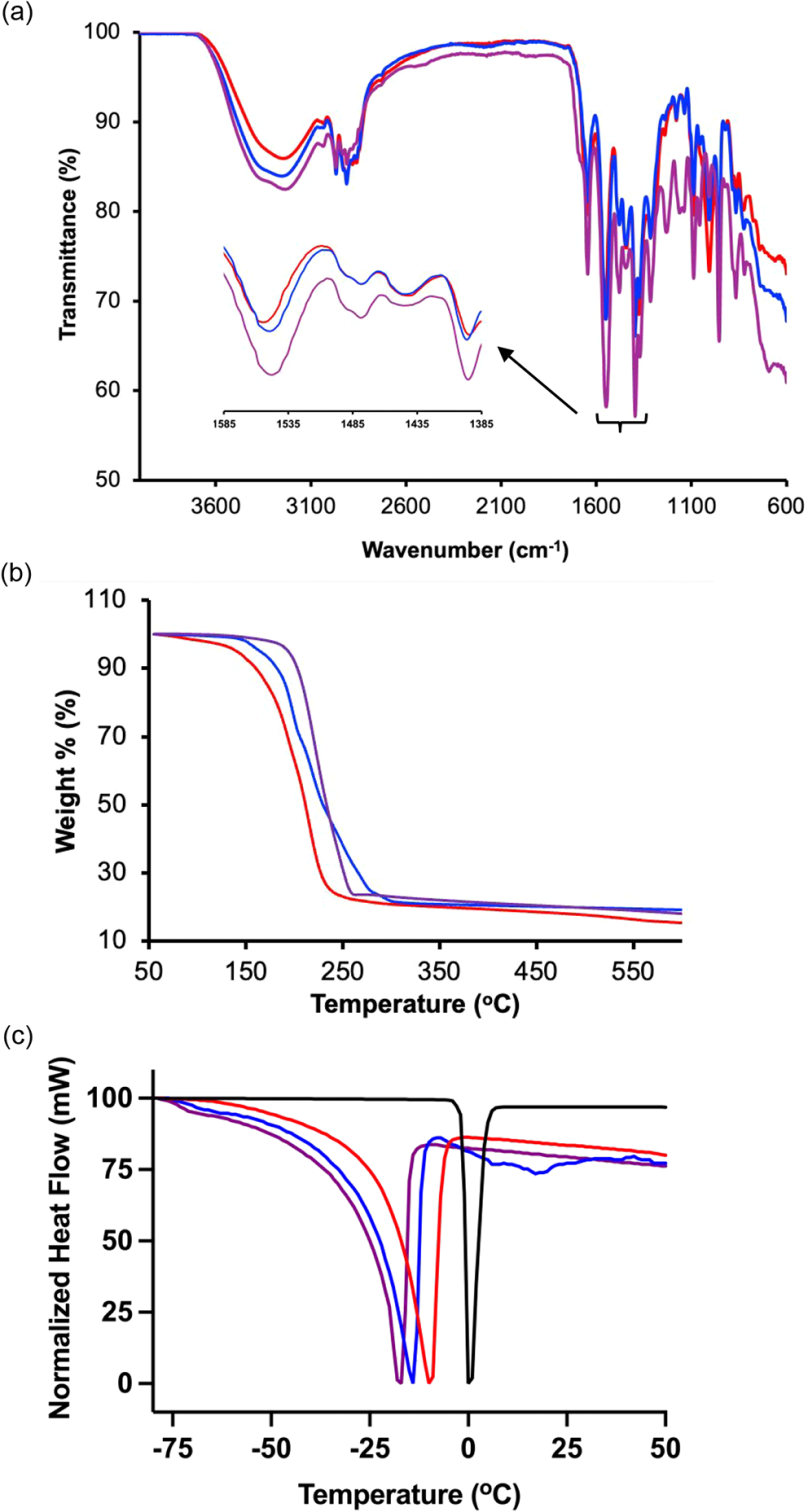
(a) Attenuated total reflectance infrared spectra and (b) thermogravimetry thermograms of bulk ILHSs. (c) Differential scanning calorimetry thermograms of ILHSs containing 30% deionized water. Blue is **1**, red is **2**, and purple is **3**.

Again, the carboxylate asymmetric stretching band originating from geranate ion in the ILHS blue-shifted from 1549 cm^–1^ in **1** to 1556 cm^–1^ in **2** upon replacing farnesol with geraniol (Figure 2a inset), further indicating that H-bonding increases with hydrophobicity. Remarkably, the difference (Δ*ν*) between the asymmetric and symmetric stretching modes of the carboxylate group in geranate can provide insight into its H-bonding and ionic interactions.^76–81^ A Δ*ν* value greater than 200 cm^–1^ indicates carboxylates, such as geranate, engaged in purely ionic interactions, while a value lower than 200 cm^–1^ suggests contributions from H-bonding interactions.^76–81^ The ATR-IR spectroscopic analysis of geranate in all ILHSs revealed that the Δν values were consistently below 200 cm^–1^ (**1**: Δ*ν* = 153 cm^–1^; **2**: Δ*ν* = 160 cm^–1^; **3**: Δ*ν* = 152 cm^–1^) (Figure 2a insert). The observed Δ*ν* value implies that geranate does not exist in a purely ionic state in these ILHSs but rather engages in substantial H-bonding interactions. Strikingly, the Δ*ν* value of **1** was lower than that of **2**, implying that the geranate in **1** engages in a H-bonding interaction slightly stronger than that in **2**, as suggested by the DFT calculations. Since both ILHSs share the same HBA and HBD but differ only in the hydrophobicity of their molecular solvent, the enhanced H-bonding interaction in **1** likely resulted from the enhanced hydrophobicity of farnesol. The similarity in Δ*ν* values of **1** and **3** suggests that both farnesol and geranic acid contribute comparably to the H-bond stabilization of the geranate, further underpinning hydrophobicity as a property to control H-bonding in ILHSs.

While ATR-IR spectroscopy can directly probe H-bonding, TGA and DSC are complementary techniques that can indirectly ascertain the extent of H-bonding in materials by examining thermal properties.^82–85^ The DSC and TGA data (Figures 2b,c, and S10–S16) concurred with those from ATR-IR, showing that increasing hydrophobicity bolsters H-bonding in the ILHSs. For example, TGA showed decreased thermal stability when farnesol in **1** was replaced with geraniol to form **2** (Figures 2b and S10). Specifically, the temperature at which the significant weight loss begins (Figures 2b and S10) corresponds to the onset of vaporization and was higher for **1** than for **2**. The onset temperature positively correlates with thermal stability. Typically, H-bonding increases the cohesive energy between molecules, enhancing thermal stability and altering the melting transition (*T*_m_), properties measurable by TGA and DSC, respectively.^84,85^ Therefore, it is reasonable to assert that increased hydrophobicity in **1** enhances H-bonding to increase thermal stability. A caveat, however, is that the long alkenyl chain in farnesol in **1** compared to that in geraniol in **2** can also promote hydrophobic interactions to bolster cohesive energy, enhancing thermal stability. To decouple the role of the alkenyl chain length, we compare the thermal stability of **2** with its structural analog, **3**. The TGA thermogram shows **3** with the same alkenyl chain length as **2**, but stronger H-bonding ability had superior thermal stability (Figure 2b). While both H-bonding and hydrophobic interactions may contribute to enhanced thermal stability, the stronger H-bonding in **3** vs **2** (Figure 2b) suggests that H-bonding plays a more dominant role in thermal stability. Nonetheless, we acknowledge that the increasing alkenyl chain length may act synergistically with H-bonding to enhance thermal stability and propose that future calorimetric and spectroscopic studies could further resolve their contributions.

To further understand whether increasing the alkenyl chain length positively correlates with H-bonding, we used DSC to determine the *T*_m_ of the ILHS-bound freezable water. Previously, bound freezable water (H-bonded water within a material, such as a polymer matrix) has been used to probe the degree of H-bonding in materials.^86–88^ Characteristically, a *T*_m_ lower than the melting point (0 °C) of bulk water suggests enhanced H-bonding in water, and this *T*_m_ negatively correlates with enhanced H-bonding within the material.^86,87^ Here, the *T*_m_ of ILHS-bound water decreased in the following order: **2** > **1** > **3** (Figures 2c and S11–S17), complementing DFT calculations and ATR-IR data, suggesting that increased hydrophobicity boosts H-bonding interactions.

Next, we hypothesize that the long farnesol alkenyl chain increases the size of the nonpolar domains, confining the polar groups into a few orientations within the polar domains to strengthen H-bonding. To test this hypothesis and precisely investigate the effect of the alkenyl chain length on ILHSs’ domain size, we used small-angle X-ray scattering (SAXS) to detect the presence and size of nanodomains in the bulk ILHSs. In SAXS, the position of the primary scattering peak (*q*) inversely correlates with the *d*-spacing (*d*), the distance between ordered domains, such as polar and nonpolar.^89^ The SAXS experiments confirmed the existence of phase-separated nanodomains in **1**–**3**, with **1** having a *d*-spacing of 3.69 ± 0.17 nm that was significantly higher than those of **2** (*d* = 3.38 ± 0.18 nm) and **3** (*d* = 2.42 ± 0.26 nm) (Figure 3a). The small *d*-spacing of **3** is attributable to the stronger attractive H-bonding ability of geranic acid compared with the alcohols in **1** and **2**. Presumably, a stronger H-bond can shorten the distance between the phase-separated domains, reducing the *d*-spacing. Therefore, the larger *d*-spacing of **1** compared with that of **2** may be counterintuitive because of the enhanced H-bonding in the former. Possibly, the larger *d*-spacing in **1** likely arises from the long alkenyl chain of farnesol extending the distance between the nonpolar and polar domains. To gain insight into contributions from the long alkenyl chain versus H-bonding in **1** relative to **2**, we use water to indirectly assess the H-bond strength. Adding water to the ILHSs can disrupt the H-bonding within the polar domain, expanding *d*-spacing or disorganizing the periodicity of the ordered domains, eliminating the SAXS peak. We expect the ILHS with the weakest H-bonding to be the most susceptible to water-induced disruption. Indeed, this experiment shows that adding 10% of water to the ILHS completely disrupted the ordering of domains in **2**, eliminating the peak, but expanded the *d*-spacing in **1** by 4.34% and **3** by 3.31%. While alkenyl chain length may likely contribute to domain spacing, the persistence of an expanded ordered domain in **1** compared with a disrupted domain in **2** under aqueous conditions suggests a correlation with enhanced H-bonding strength. Together, these findings point to a possible synergistic relationship between the molecular structure and interactions in governing nanoscale organization in multicomponent solvents.

**Figure 3:**
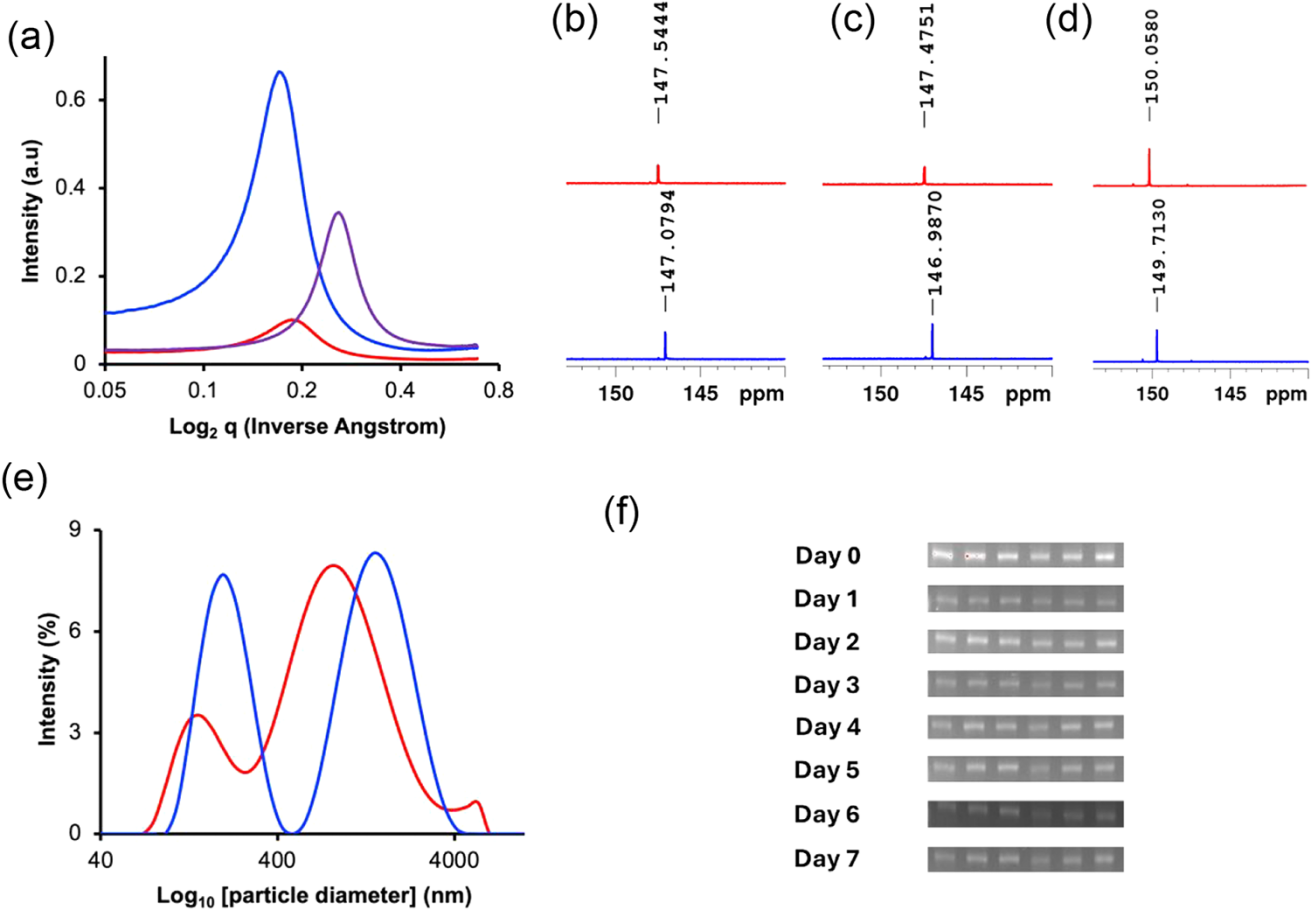
(a) Small-angle X-ray diffractograms of bulk ILHSs (blue: **1**; red: **2**; and purple: **3**) showing change in the position of the primary scattering peak (*q*), which inversely correlates with *d*-spacing. (b–d) Variable-temperature (top spectra: 0 °C; bottom spectra: 25 °C) ^13^C NMR spectra of (b) **1**, (c) **2**, and (d) **3**. (e) Dynamic light scattering shows the multimodal size distribution of a 50 mM ILHS micellar solution in water, represented by **2**. The size of the micelles increases in the presence of mCherry protein. Red: size distribution of micelles in water containing mCherry protein; Blue: size distribution of micelles in water containing mCherry protein. (f) Agarose gel electrophoresis band showing the molecular integrity of mCherry RNA in water over 7 days. From left to right, lane 1 is mCherry RNA solution in water at room temperature, lane 2 is mCherry RNA solution in water at 4 °C, lane 3 is mCherry RNA solution in water at −80 °C, lane 4 is 50 mM of **2** containing mCherry RNA, lane 5 is 50 mM of **1** containing mCherry RNA, and lane 6 is 50 mM of **3** containing mCherry RNA.

We also performed variable-temperature ^13^C nuclear magnetic resonance (VT ^13^C NMR) spectroscopy at 25 and 0 °C in CDCl_3_ to probe the degree of H-bonding in these ILHSs. VT ^13^C NMR spectroscopy provides insight into the degree of H-bonding interactions in molecular and macromolecular entities.^90–101^ The chemical shift of a carbon, such as a carboxylate carbon, is sensitive to H-bond-induced changes in electron density, indirectly probing the strength of the H-bonding interactions. As the temperature changes, H-bonds weaken or strengthen, affecting the electron density around carbon nuclei bonded to an HBA or HBD, resulting in chemical shifts that reflect the strength of H-bonding interactions. The strength of the H-bonding dictates the magnitude of the shift (Δδ), with stronger H-bonds inducing more minor shifts due to a lesser likelihood of breaking as temperature increases. Our results indicate that the β-carbon in the geranate ion exhibited a more pronounced chemical shift variation with temperature than geranate carbonyl carbon, prompting us to use this carbon to probe the extent of H-bonding in the ILHSs (Figure 3b–d). The β-carbon is within the conjugated system in the geranate ion (Figure 1a–c) and should experience structural and electronic perturbations triggered by changing H-bonding interactions. In this study, all of the ILHSs exhibited slight, but consistent, temperature-dependent variations in the β-carbon chemical shift, specifically a downfield chemical shift at 0 °C relative to 25 °C (Figure 3b–d). The magnitude of the downfield shift (**1**: Δδ = 0.4675 ± 0.0040; **2**: Δδ = 0.5159 ± 0.0390; **3**: Δδ = 0.2861 ± 0.0830) implies that **1** exhibited a stronger H-bonding interaction than **2**, corroborating data from ATR-IR, DSC, TGA, and SAXS experiments. The small Δδ in all ILHSs suggests that their H-bonding networks were relatively strong and stable, being less susceptible to temperature variation under these experimental conditions. Perhaps this stability may contribute to the limited temperature-dependent responsiveness of the chemical shift of the geranate carbonyl carbon. We expected temperature-perturbed H-bonding to the geranate oxygens to influence chemical shifts of the geranate carbonyl and β-carbons. However, charge delocalization between the two oxygens of the geranate could reduce the direct impact of temperature variations, especially in a strongly H-bonded network.

The presence of nanodomains in the ILHSs implies an ability to nanoconfine other molecules, a property with implications for diverse applications, including drug delivery and stabilization. Indeed, choline-based ILs and ILHSs have been used to deliver and stabilize biologics, including protein-based drugs.^8,9,11,30^ However, whether these novel ILHSs, **1** and **2**, can nanoconfine these drugs within their nanodomains remains unknown. We address this conundrum using dynamic light scattering to measure the hydrodynamic diameter of the ILHS micelles in water alone and water containing the mCherry protein. The ILHSs are expected to nanoconfine the polar mCherry protein within their polar domains, increasing their hydrodynamic volume. In water alone, all of the ILHSs feature a multimodal distribution comprising micelles ranging in diameter from 100–4000 nm (Figure 3e). In water containing the mCherry protein, the multimodal distribution remains, but the micellar size, corresponding to the hydrodynamic volume, increases, indicating that the ILHSs nanoconfined the mCherry protein (Figure 3e). Confinement within the ILHS domain has implications for stabilizing biological systems from degradation. Therefore, we investigated the ability of the ILHSs to stabilize mCherry RNA at room temperature. Indeed, several choline-based ILs are emerging platforms for stabilizing nucleic acids.^30,102,103^ In this experiment, the RNA fragment was incubated in the bulk ILHSs at room temperature, and agarose gel electrophoresis was used to assess the fragment degradation over 7 days. The gel band (Figures 3f and S18) shows that the RNA stored in the ILHSs at room temperature retained its molecular integrity comparable to that stored in PBS at 4 or −80 °C but better than that stored in PBS at room temperature, which had decreased band intensity after day 5. The ILHSs’ ability to preserve the RNA’s integrity is likely due to the confinement within the domains, limiting interaction with ribonucleases, which are ubiquitous in the environment. Currently, RNAs are stored at low temperatures, 4 to −80 °C; therefore, a room temperature storage system based on ILHSs will likely advance RNAs’ scientific and biomedical applications.

To conclude, we elucidated a previously unrecognized structure–property relationship in ILHSs, where increasing molecular hydrophobicity enhances the cooperative H-bonding between the components. DFT calculations, ATR-IR spectroscopy, TGA, DSC, and VT-^13^C NMR spectroscopy collectively support this finding, revealing that hydrophobic interactions promote extensive H-bonding. This emergent behavior improves nanoscale organization and enables RNA stabilization at room temperature for up to 7 days, underscoring the potential of ILHSs in RNA vaccine technologies. These insights advance our understanding of intermolecular dynamics in ILHSs and offer guiding principles for designing functional biocompatible solvents for catalysis and drug delivery applications.

## Supplementary Material



## Notes

^1^H, ^13^C, and HSQC NMR spectra for all ILHS, including their DSC and TGA thermograms and photographs of gel electrophoresis of RNA stored in ILHS (PDF)
