# Supplementary material for: Hydrophobicity-Driven Enhancement of Hydrogen Bonding in Ionic Liquid Hybrid Solvents
Source: J Phys Chem B. 2025 Jul 4;129(28):7228–37. doi: 10.1021/acs.jpcb.5c01687 (PMC12278214; doi:10.1021/acs.jpcb.5c01687)
Supplement: Supplementary file 1 [file jp5c01687_si_001.pdf]

Hydrophobicity-Driven Enhancement of Hydrogen Bonding in Ionic Liquid Hybrid Solvents

Samuel Abidemi Oluwole,[1#] Welday Desta Weldu,[1#] Christian Agatemor[1,2,*]

[1]Department of Chemistry, University of Miami, Coral Gables, FL 33146, USA.

[2]Department of Chemistry, Bucknell University, Lewisburg, Pennsylvania 17837, United States.

[#]Co-first authors

[*]Corresponding author: ca024@bucknell.edu

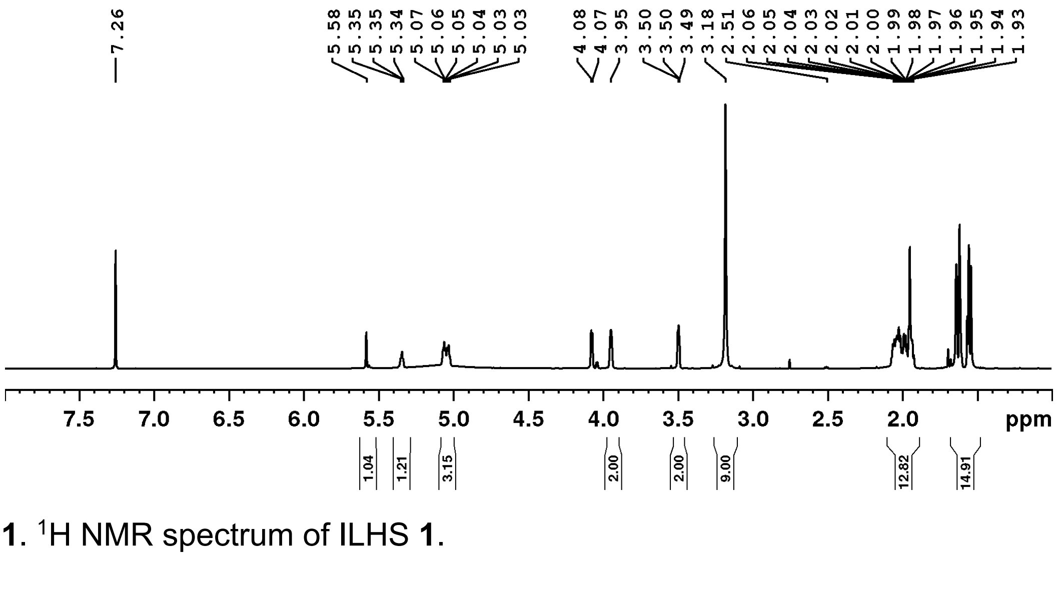

Figure S1. ^{1}H NMR spectrum of ILHS **1**.

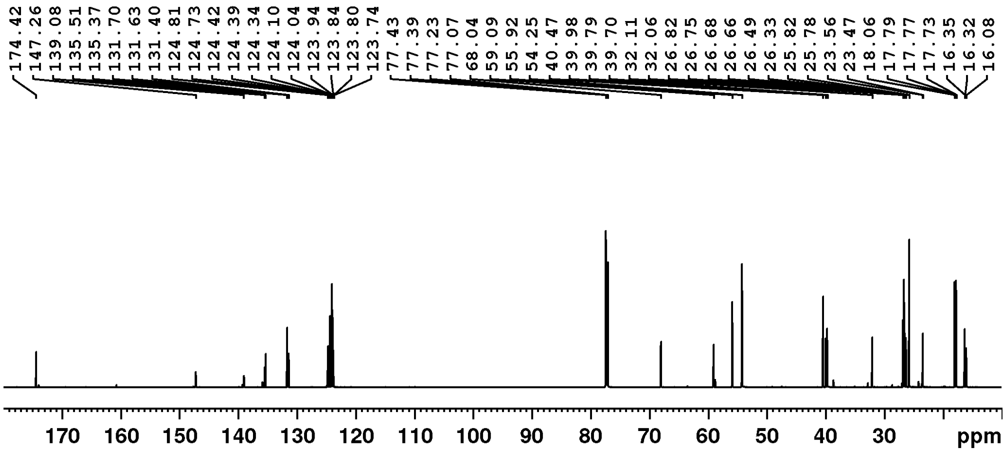

Figure S2. ^{13}C NMR spectrum of ILHS **1**

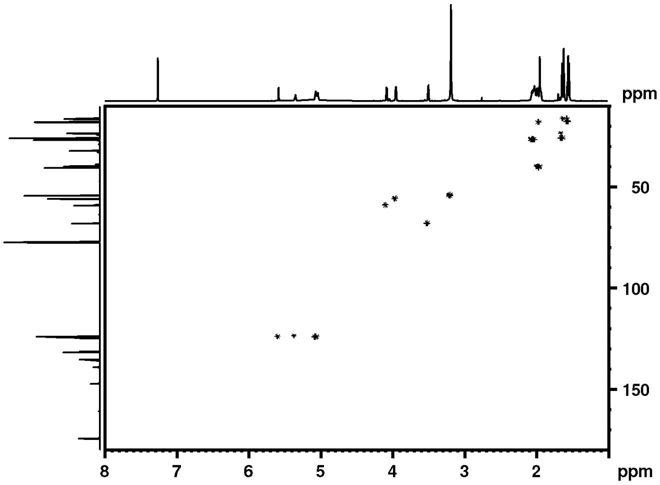

Figure S3. HSQC NMR Spectrum of ILHS **1.**

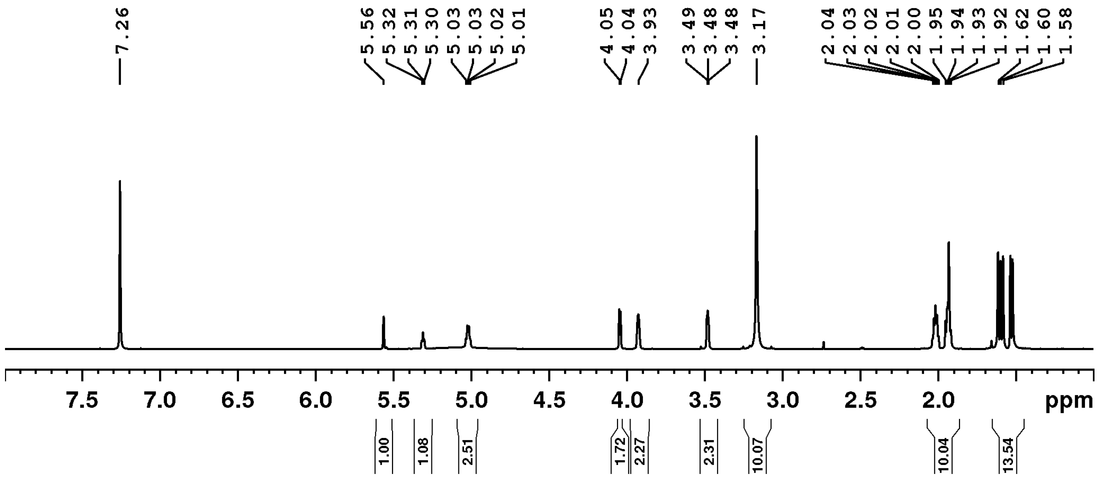

Figure S4. ^{1}H NMR spectrum of ILHS **2**.

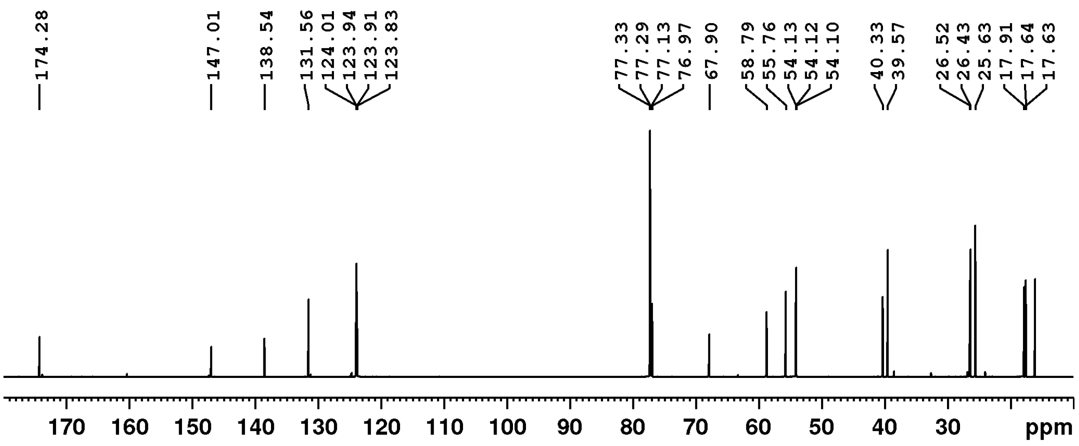

Figure S5. ^{13}C NMR spectrum of ILHS **2**.

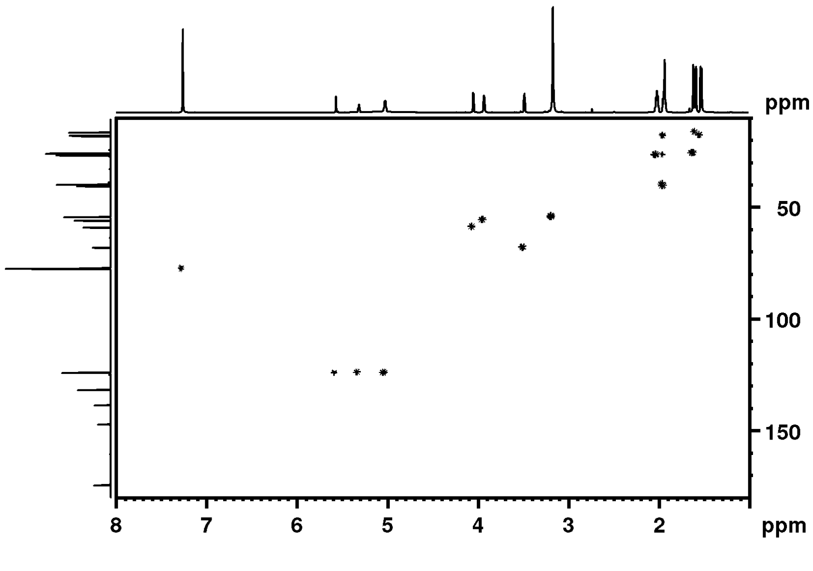

Figure S6. HSQC NMR Spectrum of ILHS **2**.

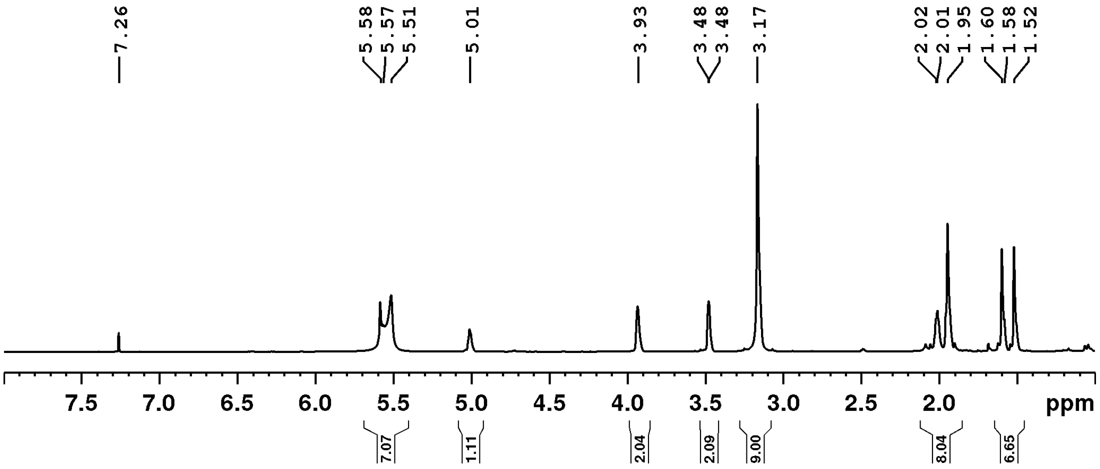

Figure S7. ^{1}H NMR spectrum of ILHS **3**.

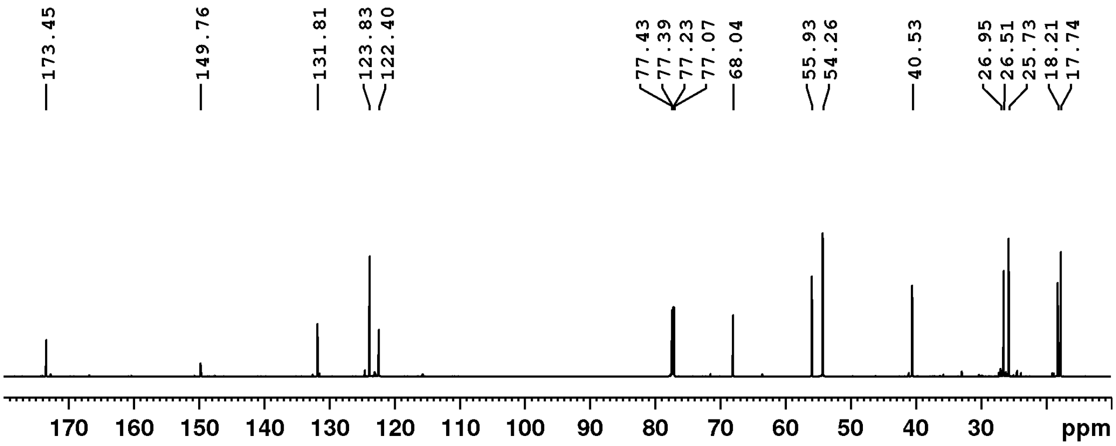

Figure S8. ^{13}C NMR Spectrum of ILHS **3**.

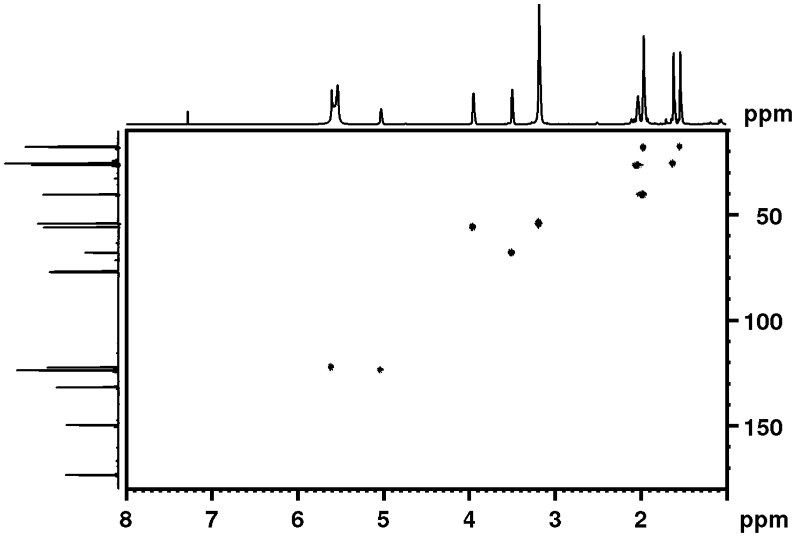

Figure S9. HSQC NMR Spectrum of ILHS **3**.

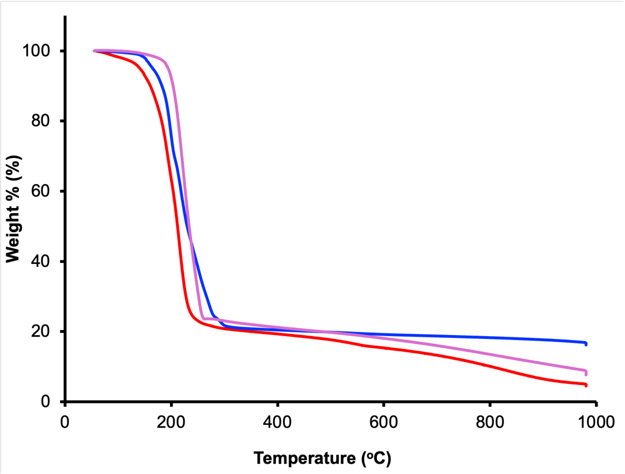

Figure S10. Thermogravimetry thermograms of the of the bulk ILHSs. Blue is **1**, red is **2** and purple is **3**.

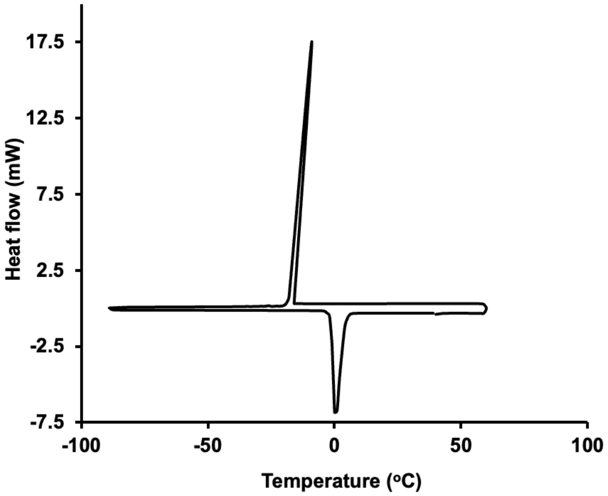

Figure S11. DSC thermogram of water.

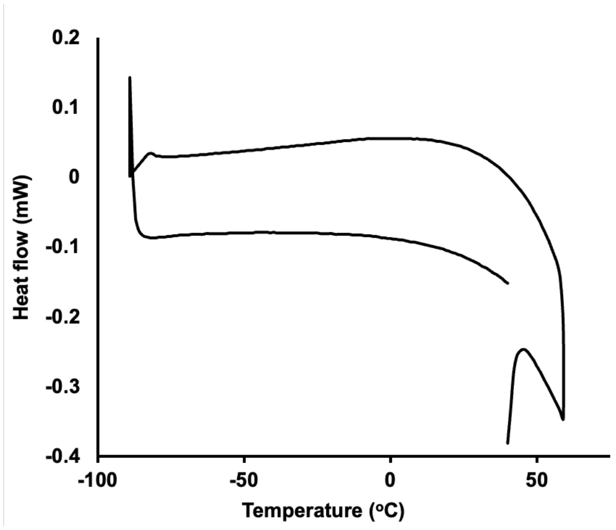

Figure S12. DSC thermogram of neat ILHS **1**.

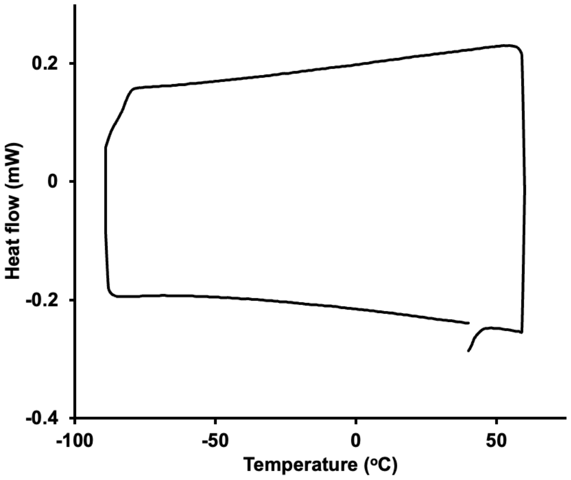

Figure S13. DSC thermogram of neat ILHS **2**.

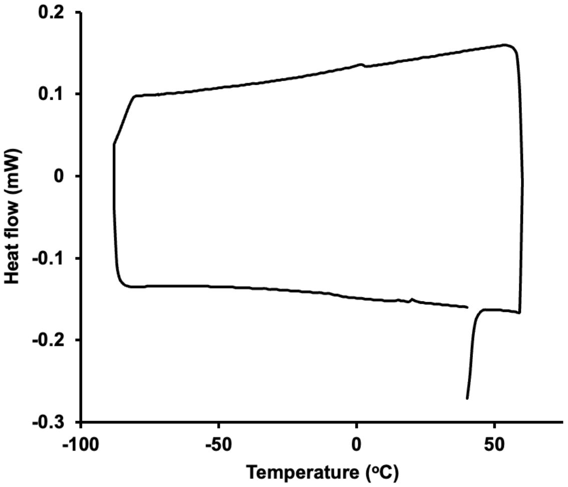

Figure S14. DSC thermogram of neat ILHS **3**.

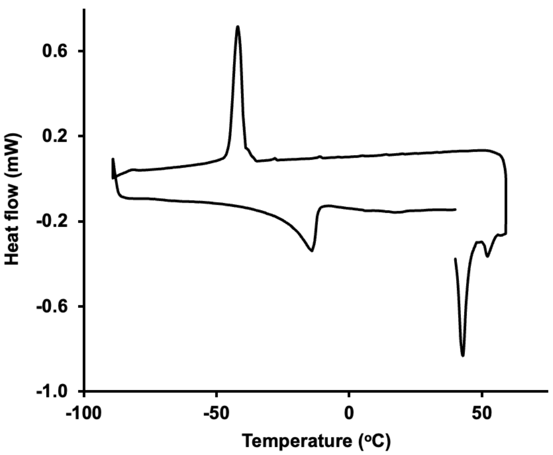

Figure S15. DSC thermogram of 30% water in ILHS **1**.

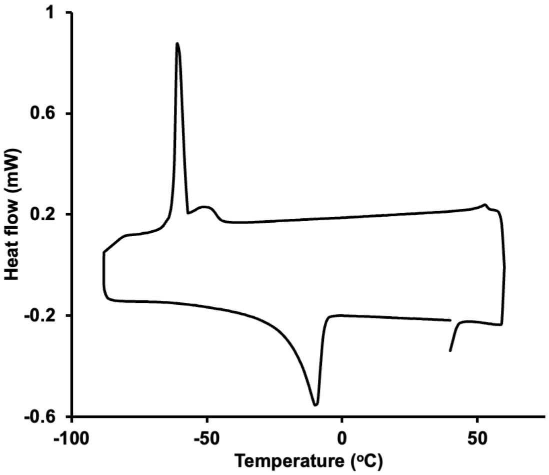

Figure S16. DSC thermogram of 30% water in ILHS **2**.

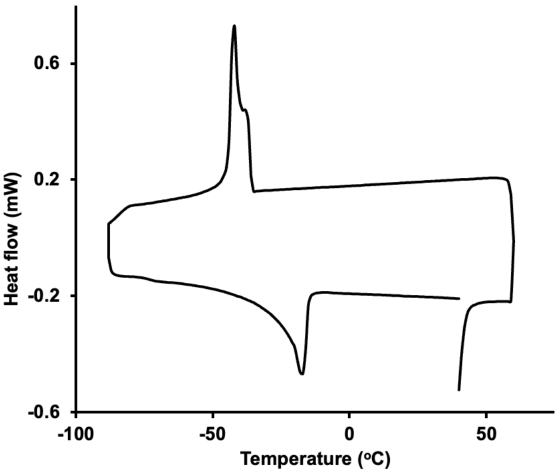

Figure S17. DSC thermogram of 30% water in **3**.

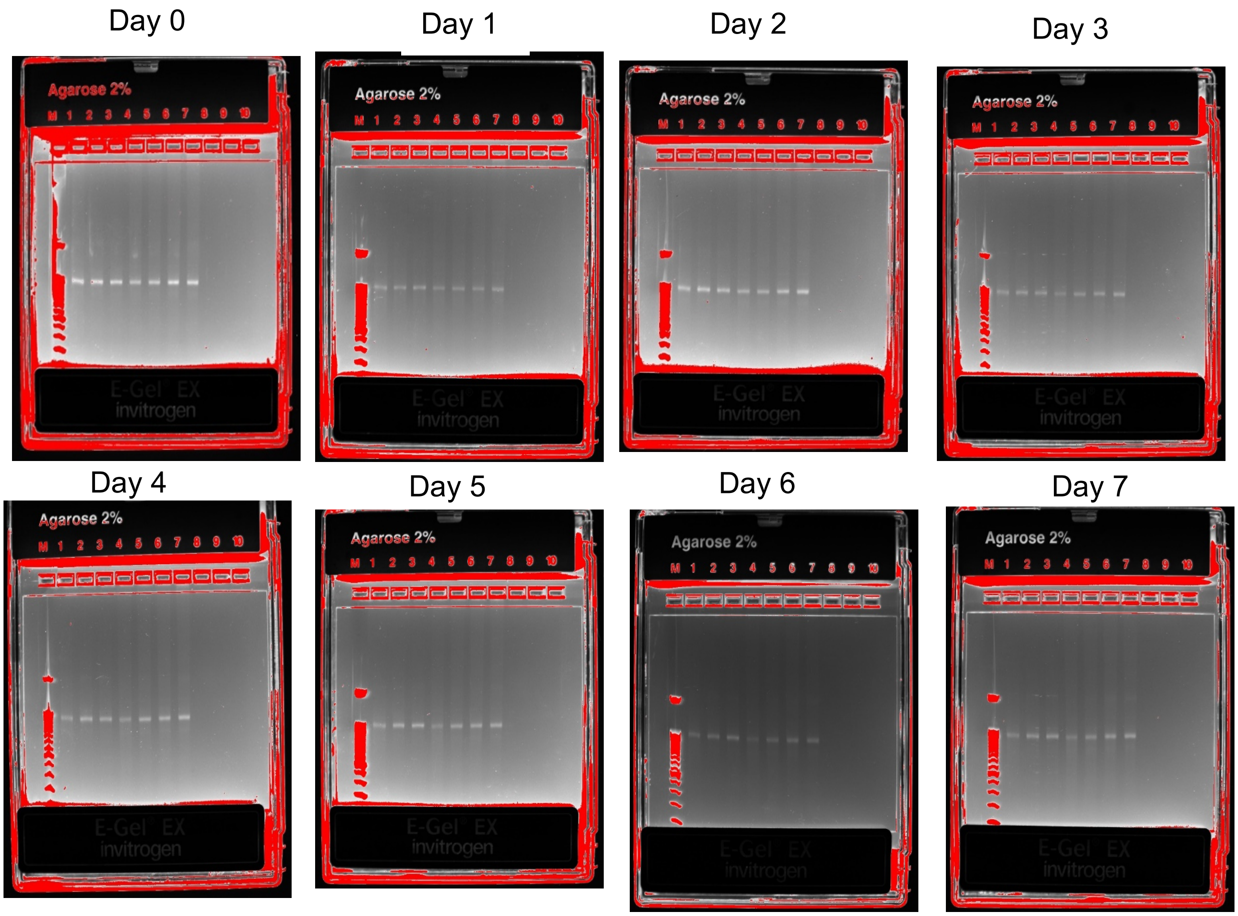

Figure S18. Gel band showing integrity of mCherry RNA. Lane M is the marker, lane 1 is mCherry RNA solution in water at room temperature, lane 2 is mCherry RNA solution in water at 4 °C, lane 3 is mCherry RNA solution in water at –80 °C, lane 4 is 50 mM of **2** containing mCherry RNA, lane 5 is 50 mM of **1** containing mCherry RNA, and lane 6 is 50 mM of **3** containing mCherry RNA; lane 7 is 50 mM of **3** containing mCherry RNA (repeat).
